# Rapid clearance of heavy chain-modified hyaluronan during resolving acute lung injury

**DOI:** 10.1186/s12931-018-0812-1

**Published:** 2018-05-31

**Authors:** Kevin Ni, Amar Gill, Victor Tseng, Andrew M. Mikosz, Kengo Koike, Erica L. Beatman, Cassie Y. Xu, Danting Cao, Fabienne Gally, Kara J. Mould, Karina A. Serban, Kelly S. Schweitzer, Keith L. March, William J. Janssen, Eva Nozik-Grayck, Stavros Garantziotis, Irina Petrache

**Affiliations:** 1 Department of Medicine, National Jewish Health, 1400 Jackson Street, Molly Blank Building, J203, Denver, CO 80206 USA; 2 Department of Biochemistry and Molecular Biology, Indiana University School of Medicine, Indianapolis, IN USA; 3 Department of Pediatrics, University of Colorado School of Medicine, Aurora, CO USA; 4 Department of Medicine, University of Colorado School of Medicine, Aurora, CO USA; 5 Department of Pathology, University of Colorado School of Medicine, Aurora, CO USA; 6 Department of Biomedical Research, National Jewish Health, Denver, CO USA; 7 Department of Medicine, University of Florida College of Medicine, Gainesville, FL USA; 8 National Institute of Environmental Health Services, Durham, NC USA

**Keywords:** Extracellular matrix, Hyaluronic acid, Inter-alpha-inhibitor, Serum-derived hyaluronan-associated protein, TNFα stimulated gene 6, Lung inflammation, Lipopolysaccharide, Pseudomonas aeruginosa

## Abstract

**Background:**

Several inflammatory lung diseases display abundant presence of hyaluronic acid (HA) bound to heavy chains (HC) of serum protein inter-alpha-inhibitor (IαI) in the extracellular matrix. The HC-HA modification is critical to neutrophil sequestration in liver sinusoids and to survival during experimental lipopolysaccharide (LPS)-induced sepsis. Therefore, the covalent HC-HA binding, which is exclusively mediated by tumor necrosis factor α (TNFα)-stimulated-gene-6 (TSG-6), may play an important role in the onset or the resolution of lung inflammation in acute lung injury (ALI) induced by respiratory infection.

**Methods:**

Reversible ALI was induced by a single intratracheal instillation of LPS or *Pseudomonas aeruginosa* in mice and outcomes were studied for up to six days. We measured in the lung or the bronchoalveolar fluid HC-HA formation, HA immunostaining localization and roughness, HA fragment abundance, and markers of lung inflammation and lung injury. We also assessed TSG-6 secretion by TNFα- or LPS-stimulated human alveolar macrophages, lung fibroblast Wi38, and bronchial epithelial BEAS-2B cells.

**Results:**

Extensive HC-modification of lung HA, localized predominantly in the peri-broncho-vascular extracellular matrix, was notable early during the onset of inflammation and was markedly decreased during its resolution. Whereas human alveolar macrophages secreted functional TSG-6 following both TNFα and LPS stimulation, fibroblasts and bronchial epithelial cells responded to only TNFα. Compared to wild type, *TSG-6*-KO mice, which lacked HC-modified HA, exhibited modest increases in inflammatory cells in the lung, but no significant differences in markers of lung inflammation or injury, including histopathological lung injury scores.

**Conclusions:**

Respiratory infection induces rapid HC modification of HA followed by fragmentation and clearance, with kinetics that parallel the onset and resolution phase of ALI, respectively. Alveolar macrophages may be an important source of pulmonary TSG-6 required for HA remodeling. The formation of HC-modified HA had a minor role in the onset, severity, or resolution of experimental reversible ALI induced by respiratory infection with gram-negative bacteria.

**Electronic supplementary material:**

The online version of this article (10.1186/s12931-018-0812-1) contains supplementary material, which is available to authorized users.

## Background

The extracellular matrix actively participates in inflammatory signaling, tissue remodeling, and repair of various tissues. A better understanding of how components of the extracellular matrix participate in acute lung injury (ALI) and repair may provide new therapeutic targets for clinical conditions such as acute respiratory distress syndrome. Hyaluronic acid (HA) is an unsulfated glycosaminoglycan (extracellular matrix polysaccharide with repeating disaccharide unit) that can be covalently modified with the heavy chains (HC) of serum protein inter-alpha-inhibitor (IαI) during inflammation [1–5], in a reaction exclusively mediated by tumor necrosis factor α (TNFα)-stimulated gene-6 (TSG-6) protein (Additional file 1). Building on reports that TSG-6 mediated formation of HC-modified HA is critical to neutrophil sequestration in liver sinusoids [6, 7] and an important protective mediator of survival in lipopolysaccharide (LPS) models of sepsis [6, 8–10], we investigated the role of HC-HA in ALI induced by respiratory infections, modeled by LPS or bacteria.

HC-modified HA is prominently featured in the extracellular matrix of chronic lung diseases such as pulmonary arterial hypertension [11], asthma [12], cystic fibrosis [13], and idiopathic pulmonary fibrosis [14], that share lung inflammation in their pathogenesis, suggesting that HC-HA formation is important in either promoting the onset or delaying the resolution of lung inflammation. We therefore used self-resolving models of ALI that were induced by intratracheal instillation of LPS or gram negative bacteria *Pseudomonas aeruginosa* (PA) to study the kinetics and role of HC-modified HA in acute lung inflammation. In addition to using *TSG-6-*knockout (KO) mice to address the functional role of HC-HA formation in ALI, we describe that LPS and *PA* induce extensive HC-HA formation during the initial phase of injury, followed by fragmentation and clearance of HC-modified HA.

### Materials and reagents

All materials and reagents used were from ThermoFisher (Waltham, MA, USA) unless otherwise specified. Reagents from Gibco (ThermoFisher) were used for cell culture.

### Mice

All animal experiments in this paper were approved by the Institutional Animal Care and Use Committee (IACUC) at National Jewish Health. *TSG-6*-KO mice (BALB/c background) were originally generated by Dr. Katalin Mikecz [15]. Studies were conducted using sex- and age matched *TSG-6*-KO mice and wild type (WT) and heterozygous (HT) littermate controls.

### Murine acute lung injury models

*Escherichia coli (E. coli)* LPS (20 μg LPS in 50 μL phosphate buffered saline (PBS), L2880, MilliporeSigma, Burlington, MA, USA) or gram negative *PA* bacteria (2 × 10^6^ CFU, colony forming unit, PA01 strain) was instilled directly into the tracheas of 10–12 week old mice using a 22-gauge oral gavage needle (7920, Cadence Science, Cranston, RI, USA) with its distal 0.5 cm end bent 40° to facilitate tracheal insertion. *PA* was provided by Dr. Kenneth Malcolm (National Jewish Health) and originally obtained from *Pseudomonas* Genetic Stock Center (East Carolina University) [15]. *PA* was grown in Luria-Bertani broth (LB), and *PA* in the exponential phase of growth was centrifuged and resuspended in 50 μL PBS for instillation. CFU was confirmed by plating dilutions of *PA* on LB agar. Mice weight was assessed every 24 h, for up to 6 days post-instillation.

## Methods

### Lung HC-HA

HC-HA formation in lung tissue was measured as described previously [16] with minor modifications. Briefly, equal mass (50 mg) of flash frozen mouse lung tissue was homogenized in PBS for 3 min using Mini-Beadbeater-16 (Biospec, Bartlesville, OK, USA) and treated with 1 U of *Streptomyces* hyaluronidase (389,561, MilliporeSigma) or PBS control for 45 min at 4 °C with mechanical agitation. Samples were then centrifuged (13,000 *g*, 5 min, 4 °C) and supernatants were then incubated (37 °C; 30 min) with mechanical agitation. The samples were then combined with Laemmli Buffer, separated by SDS-PAGE (Stain-free Criterion TGX 7.5% gels, Biorad, Hercucles, CA, USA) and transferred to Immobilon-P PVDF membrane (MilliporeSigma) using TransBlot Semi-Dry (Biorad). The western blot was probed using rabbit-anti-hIαI antibody (A0301, DAKO, Agilent, Santa Clara, CA, USA), which has been validated for detecting mouse IαI and HC-HA formation in injured mouse lung [17]. ChemiDoc MP (Biorad) was used to image the Stain-free gels for total protein. Densitometry was performed using Image Studio Lite (Licor, Lincoln, NE, USA).

### Messenger RNA (mRNA) studies

Total ribonucleic acid (RNA) was extracted from cultured cells using RNeasy Mini Plus (Qiagen, Germantown, MD, USA) and from whole lung using Trizol Plus RNA Purification Kit with on-column deoxyribonuclease (DNAse) digest performed using PureLink DNase. Mouse lung was homogenized in Trizol using Mini-Beadbeater-16 (Biospec); 1000–2000 ng of total extracted RNA was used to synthesize complementary DNA (cDNA) (High-Capacity cDNA Reverse Transcription). Real-time quantitative polymerase chain reaction (qPCR) was performed on the StepOnePlus System using Taqman Universal PCR Master Mix and Taqman probes: *hTSG-6* (Hs01113602_m1), *msTNFα* (Mm00443258_m1), *msTSG-6* (Mm00493736_m1) [18, 19], *msHAS1* (Mm03048195_m1), *msHAS2* (Mm00515089_m1), *msHAS3* (Mm00515092_m1), *msHYAL1* (Mm00476206_m1), *msHYAL2* (Mm01230688_g1), *msTMEM2* (Mm00459599_m1), and *msCEMIP* (Mm00472921_m1). Relative mRNA expression was calculated using the double delta comparative (ΔΔCt) method and *18 s* RNA endogenous control (Taqman Hs99999901_s1).

### HA histology

Mice were euthanized by isoflurane overdose, bilateral thoracotomy, and perfusion of the lungs via the right ventricle using 10 mL of blood bank saline. LPS injured lungs were inflated with a PBS equilibrated solution containing 4% paraformaldehyde (PFA) (15,710, Electron Microscopy Sciences, Hatfield, PA, USA) and 0.33% low melting point agarose. Inflated lungs were immersion fixed overnight (24 h) in 4% PFA at 4 °C with gentle rocking and then sequentially incubated for 1 h in PBS and 4 h in PBS containing 25% sucrose and 25% optimal cutting temperature (OCT) compound. The lungs were then embedded in OCT compound and frozen using dry ice. Ten μm sections were cut using a cryostat and allowed to air dry before washing in PBS to remove OCT compound.

*PA* injured lungs were inflated with 10% neutral buffered formalin containing 0.25% low melting point agarose and then immersion fixed in 10% neutral buffered formalin overnight at room temperature before paraffin embedding and sectioning (3 μm). Paraffin embedded tissue sections were then mounted on slides and processed as follows: mounted tissues were deparafinized and rehydrated using successive incubations in xylene (3 × 5 min), 100% ethanol (2 × 5 min), 95% ethanol (2 × 5 min) and equilibration in PBS followed by water. The tissue was then placed in pressure cooker containing citric acid based antigen unmasking solution (Vector Labs, Burlingame, CA, USA) and microwaved.

Staining of lung sections was performed as follows: tissue sections were blocked using PBS solution containing 3% bovine serum albumin (BSA, MilliporeSigma) and 0.1% Triton X-100 (MilliporeSigma). Biotinylated hyaluronan binding protein (50 μg/100 μl stock, 385,911, MilliporeSigma), rabbit anti-human HC2 (NBP2–31750, Novus, Littleton, CO, USA), and rat anti-mouse CD68 (FA-11, Biolegend, San Diego, CA) were added at 1:100 and incubated overnight at 4 °C. Streptavidin Alexa Flour 488 (S-11223) was used at 1:1000. Cy3 donkey anti-rabbit (711–165-152, Jackson ImmunoResearch, West Grove, PA, USA) and Cy5 donkey anti-rat (712–175-153, Jackson ImmunoResearch) were used at 1:2000. Tissue was mounted using ProLong Gold AntiFade with DAPI and imaged using laser scanning confocal microscope 700 confocal (Zeiss, Jena, Germany).

### Histologic ALI scoring

Unlavaged mice lungs were perfused as described above. The left lung was inflated at 20 cm H_2_O with 0.25% agarose in 10% formalin and immersion fixed overnight in 10% formalin following current guidelines [20]. The fixed lung was placed in a molding box, encased in agarose, and 3 mm thick transverse pieces (apex to base) of the lung were sliced to ensure adequate sampling of the entire lung for histological scoring. The lung pieces were paraffin embedded together, sliced at 3 μm thick, and deparafinized and rehydrated as described above. The slides were stained with Harris Hematoxylin (2 min), Clarifier 1 (1 min), Bluing reagent (1 min), Eosin Y (30 s), dehydrated, and mounted. 4–5 fields (400X total magnification) of each transversely sliced lung piece (4–5 pieces total) were scored by a pathologist using the scoring system published by American Thoracic Society [20], which assigns weighted scores for five parameters of ALI injury and provides a final averaged score between 0 (no injury) and 1 (most severe).

### Cell culture

Primary human alveolar macrophages (hAM) were obtained by bronchoalveolar lavage of de-identified non-diseased human explanted lungs and enriched by 2 h attachment to tissue culture treated plastic in Roswell Park Memorial Institute (RPMI) media with 1% penicillin/streptomycin. Non-adherent cells were removed by PBS wash. Indicated treatments were performed by incubating in RPMI media with 2% fetal bovine serum (FBS, HyClone, GE Healthcare, Marlborough, MA, USA) and 1X penicillin-streptomycin and either vehicle (0.1% bovine serum albumin in PBS), 20 ng/mL tumor necrosis factor α (TNFα, R&D, Minneapolis, MN, USA), or 50 ng/mL ultrapure *E. coli* LPS (LPS-EK, InvivoGen, San Diego, CA, USA) for either 6 h or 24 h.

Peripheral Blood Mononuclear Cell Derived Macrophages (PBDM) were enriched by negative selection from whole blood using DynaBeads Untouched Human Monocyte Kit and by attachment to tissue culture plastic. Treatment with macrophage colony stimulating factor (20 ng/mL MCSF, R&D) over six days was used to differentiate PBMC into macrophage-like cells. Macrophage differentiation was performed in RPMI under serum free conditions with supplemental 1X non-essential amino acids, 1 mM sodium pyruvate, 2 mM glutamine, and 1X penicillin-streptomycin for days 1–3 and with additional 10% FBS for days 4–6. For experiments, cells were incubated in RPMI media with 2% FBS and treated with indicated stimuli for 24 h.

BEAS-2B transformed human lung bronchial epithelial cells were cultured submerged in Dulbecco’s Modified Eagle Medium (DMEM), high glucose (4500 mg/L) media with 10% FBS and 1% penicillin/streptomycin. For experiments, cells were washed once with PBS and then incubated in basal DMEM media with 2% FBS and indicated stimuli for 24 h.

Wi38 primary human fetal lung fibroblasts were cultured using Minimum Essential *Media* (MEM) media with 10% FBS and 1% penicillin/streptomycin. Cells were used between passages 8–12 for experiments, during which they were incubated with the indicated stimuli in basal MEM media with 2% FBS.

Human adipose stromal/progenitor cells (ASC) isolation, expansion, and characterization have been previously described [21–23]. Briefly, ASC were obtained by liposuction from three human donors (two abdominal and one flank lipoaspirate) and then digested using collagenase I (Worthington, Lakewood, NJ, USA) under mechanical agitation for 2 h at 37 °C and centrifuged at 300 *g* for 8 min to obtain a pellet containing the stromal vascular fraction. This fraction was filtered using 250 μm Nitex filters (Sefar America, Buffalo, NY, USA), and red blood cells were lysed using ammonium chloride potassium lysis buffer (154 mM NH_4_Cl, 10 mM KHCO_3_, and 0.1 mM ethylenediaminetetraacetic acid (EDTA)). Cells were then cultured using Endothelial Cell Growth Medium (EGM2-MV) media (Lonza, Allendale, NJ). Cells were used for experiments between passages 4–6. To stimulate TSG-6 secretion [22], ASC were washed with PBS to remove residual FBS and then incubated in basal Endothelial Basal Medium-2 (EBM2) media (Lonza) with 20 ng/mL TNFα (R&D) for 24 h. Demographic information of the ASC donors have been described previously [24].

### Human TSG-6 (hTSG-6) western blot

Conditioned media was centrifuged to remove detached cells (5 min, 600 *g*) and then mixed with Laemmli buffer. Proteins were separated by sodium dodecyl sulfate polyacrylamide gel electrophoresis (SDS-PAGE) using Stain-Free Criterion TGX 4–20% gradient gels (Biorad), transferred, and imaged similarly as HC-HA blots. The blot was probed using goat-anti-hTSG-6 antibody (AF2104, R&D).

### hTSG-6 ELISA

Conditioned media was collected and centrifuged (600 *g*, 5 min) and human TSG-6 (hTSG-6) was measured as previously described [22] using a highly sensitive sandwich ELISA developed using commercially available antibodies [25] and validated by *TSG-6* small interfering RNA (siRNA) in human MSC [25] and ASC [22]. Briefly, Nunc MaxiSorp 96-well plates were coated with rat anti-hTSG-6 antibody (A38.1.20; Santa Cruz) diluted in 0.2 M sodium bicarbonate buffer. Detection was performed using biotinylated goat anti-hTSG-6 antibody (BAF2104, R&D), Streptavidin-HRP (R&D), HRP Substrate (R&D) and quenched using 1 M H_2_SO_4_. To determine the extent of TSG-6 secretion relative to cell number, viable cell numbers were assessed by trypan blue exclusion and counted by hemocytometer. Recombinant human TSG-6 (2104-TS-050, R&D) in the absence and presence of FBS (2%) was used for standard curve (Additional file 2), since we found that the presence of FBS lowered the magnitude of HRP substrate color development. This phenomenon may be due to TSG-6 forming TSG-6-HC covalent intermediate in the presence of IαI present in the serum and may explain why efforts to directly measure TSG-6 in human serum have been particularly challenging [26].

### Time-course of macrophage expression of *TSG-6* and genes implicated in HA breakdown in LPS-challenged mice

Expression of mouse *TSG-6* (*msTSG-6)*, also known as *TNFα-induced-protein 6 (TNFAIP6*), as well as *msHYAL1–2*, *msTMEM2*, and *msCEMIP* was identified using Ensembl gene annotation data in a previously published data set. The detailed methods, pathway analysis of the RNA-sequencing (RNA-seq) data, and *National Center for Biotechnology Information* (NCBI) deposition have been described here [27]. Briefly, RNA-seq analysis was performed on bone-marrow-derived, recruited and resident macrophages isolated from bronchoalveolar lavage of intratracheal LPS treated mice (C57BL/6, 10–12 week old; 0, 3, 6, 9, and 12 dpi).

### HA fragmentation assessment in whole lung

HA fragmentation in lung tissue was assessed using a protocol generously provided by Cleveland Clinic Program of Excellence in Glycoscience. Briefly, dedicated (non-lavaged) lungs were perfused with 10 mL blood buffered saline and flash frozen. Proteinase K (1 mg/mL) resuspended in 100 mM ammonium acetate (pH 7.0) with 0.01% sodium dodecyl sulfate was used to lyse 50 mg of tissue (24 h; 60 °C). 100% ethanol was added to precipitate glycoaminiglycans and samples were washed using 75% ethanol. Samples were resuspended in 100 mM ammonium acetate, and 100 °C heat was used to inactivate Proteinase K. Overnight benzonase treatment (MilliporeSigma) was used to degrade nucleic acids, and 100 °C heat was used to inactivate benzonase. 100 and 75% ethanol was then used to precipitate and wash the samples before resuspending in 100 mM ammonium acetate. Samples were equally divided and paired, having a half of the sample left untreated, and half treated with 0.2 turbidity reducing units (TRU) of *Streptomyces* hyaluronidase (Seikagaku, amsbio, Cambridge, MA). All samples were lyophilized and resuspended in formamide (MilliporeSigma) for loading on 1% agarose gel (SeaKem HGT Agarose, Lonza). Gels were stained overnight in Stains-All (1.25 mg/200 mL in 30% ethanol), equilibrated in water, destained using light, and imaged using Cy5 695/55 epi-fluorescence filter on ChemiDoc MP [28]. Select-HA of predetermined sizes (2500, 1000, 500, and 250 kDa HA) and Select-HA HiLadder (Hyalose, Oklahoma City, Oklahoma, USA) were used to size HA fragments. Densitometry of the distribution of HA staining was performed using ImageJ as described before [29]. It has been described previously that agarose gel electrophoresis method is optimally suited for resolving high and medium molecular weight HA (> 200 kDa) and that chromatography and polyacrylamide gel electrophoresis can provide better resolution and quantification of low molecular weight HA [30, 31].

### HA staining characterization

 Confocal Z-stacks were de-identified for the experimental group, and HA staining was blindly scored. Briefly, three to five representative 320 × 320 μm images of the left lung were taken from each mouse. From each image, HA staining in the peri-broncho-vascular interstitium was sampled by taking five 9.4 × 9.4 μm representative subselection snapshots. A max intensity Z projection was then prepared using the Fiji distribution of ImageJ [32] and roughness was calculated by determining the surface area [33] of the plotted intensity of HA staining using the SurfCharJ plugin [34]. The roughness was normalized by dividing it by the average staining intensity of the snapshot. A mean normalized roughness was then determined for each mouse.

### Bronchoalveolar lavage fluid (BALF) collection and flow cytometry

Tracheotomy was used to visualize the trachea and insert an 18-gauge angiocatheter (4075, JELCO-W, Smiths-Medical, Minneapolis, MN). BALF was obtained by five serial instillations (1 × 1 mL and 4 × 0.9 mL) of PBS containing 2 mM EDTA (a return of 4 mL of total BALF was consistently obtained with this protocol). For the total CD45^+^ count, aliquots of the five lavages were combined, blocked with CD16/CD32 (clone 93, eBioscience, ThermoFisher), stained with CD45 (30-F11, BD, Franklin Lakes, NJ, USA), and mixed with 123count eBeads (eBioscience). Cells used for total cell counts were stained and fixed without any centrifugation to avoid variability introduced by pelleting and aspirating. Using the absolute concentration of the counting beads added and the ratio of total CD45^+^ events to total bead events, the concentration of CD45^+^ cells was determined and then multiplied by 4 mL to obtain total CD45^+^ counts. For BALF cellular differentials, the five lavages were combined and centrifuged, blocked with CD16/CD32 (eBioscience), and stained with CD45 (30-F11, BD), Ly6G (1A8, Biolegend), CD64 (X54–5/7.1, BD), CD11c (N418, eBioscience), F4/80 (BM8, eBioscience), CD11b (M1/70, eBioscience), Siglec-F (E50–2440, BD), CD4 (RM4–5, Biolegend), and CD8a (53–6.7, Biolegend). Flow wash buffer consisting of PBS with 9% FBS and 0.5 mM EDTA was used to resuspend and wash cells. Flow data, which included minimum of 20,000 (PBS group) and 100,000 (LPS group) CD45^+^ leukocyte events for each sample, was collected using LSR II cytometer (BD) and analyzed using Flowjo (Ashland, Oregon, USA).

### ELISA

Albumin-, receptor for advanced glycation end products (RAGE)-, and HA ELISAs were performed on the combined supernatant obtained from pelleting the first three BALF aliquots (total 2.6 mL volume). Manufacturer’s protocols were followed, using mouse albumin ELISA quantitation set (Bethyl Labs, Montgomery, TX); RAGE Duoset ELISA (R&D); HA Duoset ELISA (R&D), using sample dilutions of 1:3000; 1:6; and 1:4 (ctl group) or 1:12 (LPS group), respectively. Capture antibody coating and HRP detection were performed as described for TSG-6 ELISA.

### Statistics

Statistical significance was calculated using ANOVA and Tukey’s multiple comparison test in Prism (Graphpad, La Jolla, CA). Data points from individual mice or independent experiments were plotted unless otherwise specified. Results were considered significant at *P* < 0.05.

## Results

### Induction and clearance of HC-modified HA during ALI

**Fig. 1:**
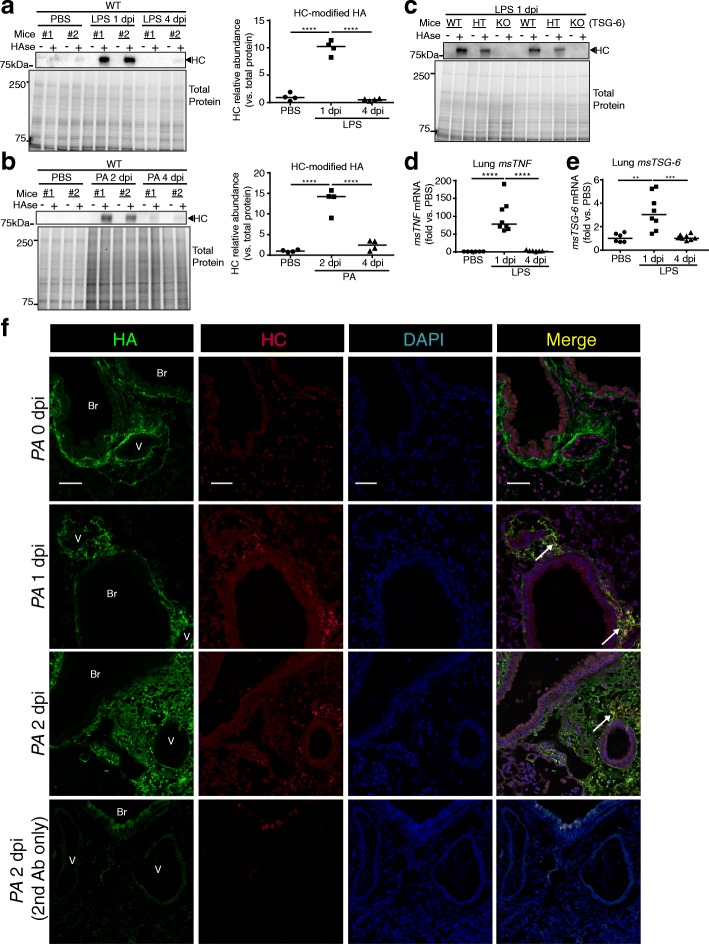
HC-HA formation after LPS or *PA* injury. **a-c**. Abundance of heavy chain (HC)-linked HA in lung lysates detected by western blot using IαI antibody (recognizing HC) on lungs before (−) and after (+) hyaluronidase (HAse), which releases HC linked to HA. Each lane represents an individual mouse lung exposed to intratracheally instilled LPS (20 μg; **a**) or *Pseudomonas aeruginosa* (*PA*, 2*10^6^ CFU; **b**) or control PBS for the indicated time, noted as days post instillation (dpi). Lung HC abundance was expressed relative to that of total protein, measured by densitometry (**a-b**). **c**. Exclusive role of TSG-6 in forming HC-HA was confirmed using wild type (WT), heterozygous (HT), and knockout (KO) for *TSG-6*. **d-e**. *msTNFα* and *msTSG-6* expression levels measured by qPCR in whole lung following LPS. Data in **a-b** and **d-e** analyzed by ANOVA with Tukey’s multiple comparisons; **P < 0.01, ***P < 0.001, ****P < .0001. **f**. Immunofluorescence images of HA and HC localization in formalin-fixed, paraffin-embedded lung sections from control (0 dpi) and *PA*-injured (1 and 2 dpi) mice, using antibodies against HA binding protein (red) or HC2 (green), and staining for nuclei with DAPI (Blue). Staining control provided in the last row, using secondary antibody only. Note HC-HA co-localization in the peri-broncho (Br)-vascular (V) interstitium (white arrow); scale bar 50 μm.

To investigate the kinetics of HC-HA formation during respiratory infection-induced ALI, we delivered LPS or *Pseudomonas aeruginosa* (*PA*) to the lungs of adult mice and then probed for HC-HA in whole lung homogenates. We first assessed HC-HA formation in lung tissue from mice exposed to LPS or PBS control, by ex vivo treating the saline perfused and homogenized lung tissue with hyaluronidase, which releases any HC linked to HA, followed by western blot detection of released HC. We observed extensive HC-HA formation in LPS-exposed lungs at one-day post instillation compared to contemporaneous PBS controls in both wild type (Fig. 1a) and *TSG-6* heterozygous mice (Additional file 3: Figure A). However, at 4 days post-LPS challenge, there was minimal HC-modified HA in the lungs of wild type (Fig. 1a) or *TSG-6* heterozygous mice (Additional file 3: Figure A). We next assessed HC-HA formation using a clinically relevant model of gram negative *PA*-bacteria-induced ALI. The extensive HC-HA formation noted in lungs 2 days post intratracheal instillation of *PA* was followed by minimal HC-modified HA at 4 days post *PA* instillation in wild type (Fig. 1a) or *TSG-6* heterozygous mice (Additional file 3: Figure B). To confirm that TSG-6 exclusively mediates HC covalent modification, we measured HC-HA in *TSG-6*-KO mice and noted no HC-HA at one-day post LPS instillation compared to wild type and heterozygous littermates (Fig. 1c).

Having shown HC-HA formation, we studied if lung levels of the eponymous TSG-6 inducer TNFα or levels of *TSG-6* mRNA paralleled those of HC-modified HA in ALI induced by LPS. Both *TNFα* and *TSG-6* were rapidly induced 1 day post LPS instillation with expression levels returning to baseline by day 4 (Fig. 1d-e). To determine the localization of HC-HA formation, lung sections from *PA*-injured lungs were stained for HA and HC. HC-modified HA determined by co-localization of HA and HC staining was absent in uninjured lungs, but was abundant in the peri-broncho-vascular interstitium following 1- and 2 days post PA instillation (Fig. 1f). These results suggest rapid formation and clearance of HC-HA during respiratory infection-induced ALI.

### TSG-6 production by lung resident cells

**Fig. 2:**
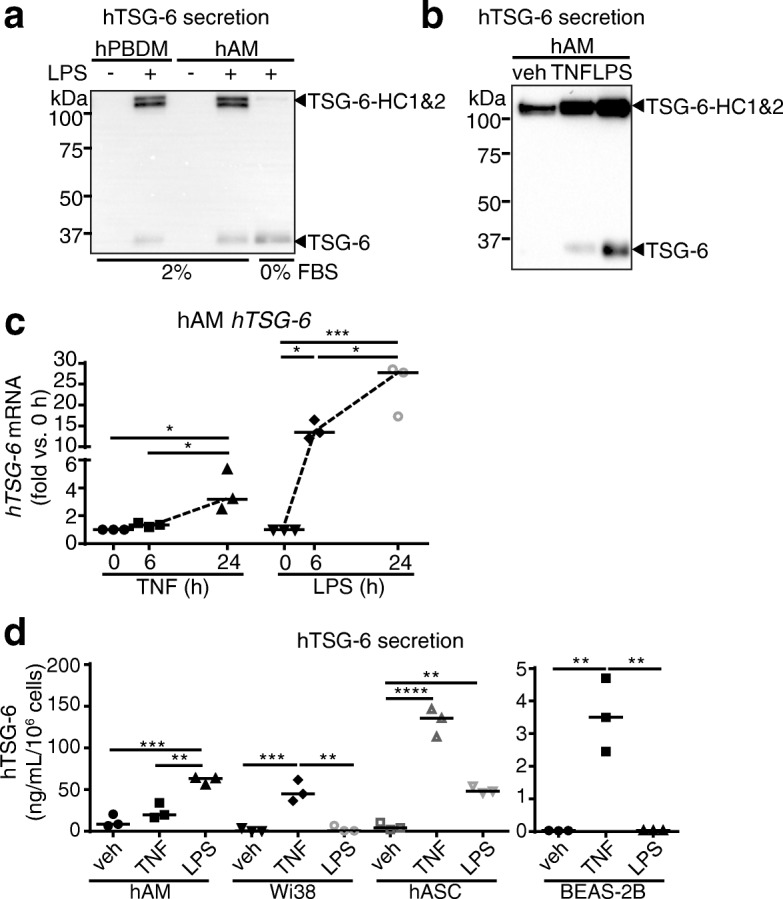
TSG-6 induction by TNFα or LPS stimulation of lung cells. **a**. Presence of TSG-6 in conditioned media of cultured human peripheral blood mononuclear cell-derived macrophages (hPBDM) and in human alveolar macrophages (hAM) following LPS stimulation (50 ng/mL, 24 h) detected by western blotting with TSG-6 antibody. Note that TSG-6 forms covalent TSG-6-HC intermediates only in the presence of 2% FBS (which contains serum inter-alpha-inhibitor that provides HC1 and HC2). **b-c**. TSG-6 secreted protein in supernatants (**b**; 2% FBS) and mRNA expression (**c**) of hAM stimulated with TNFα (20 ng/mL, 24 h or indicated time) or LPS (50 ng/mL, 24 h or indicated time), or vehicle (veh) assessed by western blot and qPCR, respectively. **d**. Levels of TSG-6 protein secreted in supernatant of hAM, human lung fibroblasts Wi38, human adipose stromal/progenitor cells (hASC), and human bronchoepithelial cells BEAS-2B stimulated with TNFα or LPS, measured by ELISA. In **c-d**, each data point represents an independent experiment; data analyzed with ANOVA and Tukey’s multiple comparisons. *P < 0.05, **P < 0.01, ***P < 0.001, ****P < .0001

To determine which lung cells produce the TSG-6 that is required for forming HC-HA, we investigated TSG-6 production by cultured lung macrophages, bronchoepithelial cells, and fibroblasts. Cells were stimulated with LPS since previous work identified it as potent stimulator of TSG-6 RNA induction and secretion by myeloid cells [35, 36]. As a first step, primary alveolar macrophage (hAM) and peripheral blood mononuclear cell derived macrophage (hPBDM) were stimulated with vehicle or LPS, then TSG-6 secretion and functionality was assessed in conditioned media by western blot in the presence of serum containing IαI (the source of HC) or in the absence of serum (as a negative control). As anticipated, both hAM and hPBDM secreted TSG-6 (35 kDa) only after stimulation with LPS (Fig. 2a). Notably, secreted TSG-6 formed covalent TSG-6-HC intermediates (130 kDa) only in the presence of serum IαI-containing FBS (Fig. 2a-b). Both LPS and TNFα, the standard and eponymous inducer of TSG-6 production, induced TSG-6 secretion in hAM (Fig. 2b**)** at levels consistent with the magnitude of mRNA induction (Fig. 2c).

When we next compared different lung cell types to adipose stromal/progenitor cell, which are known to potently secrete TSG-6 in response to TNFα, we noticed significantly different patterns of TSG-6 secretion in response to TNFα or LPS (Fig. 2d). While hAM secreted TSG-6 more robustly in response to LPS than TNFα, adipose stromal/progenitor cell had a potent response to TNFα, but similar levels of TSG-6 secretion in response to LPS as the hAM. In contrast, lung fibroblast and bronchoepithelial cells only responded to TNFα, and bronchoepithelial cells only showed comparatively minor levels of TSG-6 secretion in response to TNFα.

To validate that AM produce TSG-6 in vivo and to determine the relative difference in *TSG-6* induction in bone-marrow-derived, recruited vs. resident AM*,* we evaluated our RNA-seq database of resident and recruited alveolar macrophages isolated from bronchoalveolar lavage of LPS treated mice (previously published in [27]). We noted similar early induction of *TSG-6* mRNA that was highest at day 3 following LPS and noted generally a more robust induction in recruited- compared to resident AM (Additional file 4).

### Assessment of HA remodeling during ALI

**Fig. 3:**
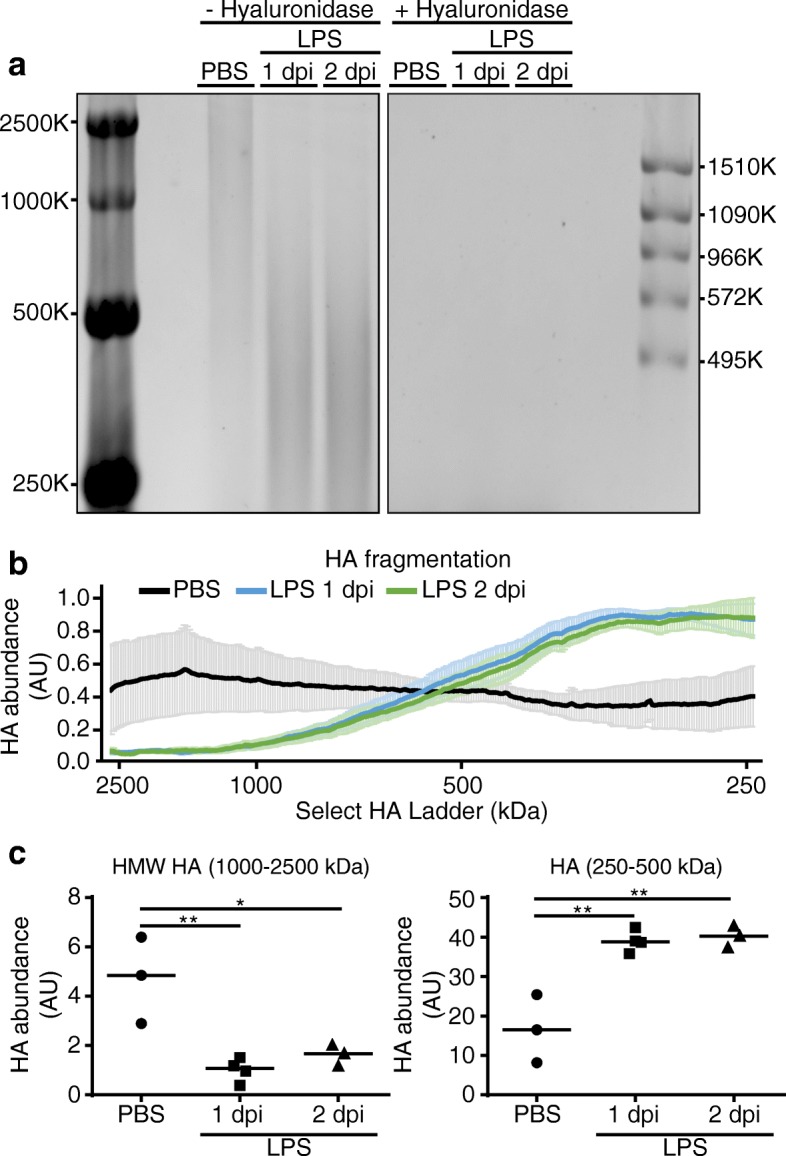
Effect of LPS on lung HA molecular weight distribution. **a**. Detection of HA by Stains-All staining of agarose gel-resolved glycosaminoglycans extracted from lung tissue of mice following intratracheal instillation of vehicle (PBS) control or LPS for the indicated time. Extracted samples were examined prior to (−) and following hyaluronidase treatment (+). Select-HA consisting of 2500, 1000, 500, and 250 kDa HA and Select-HA HiLadder consisting of 1510, 1090, 966, 572, and 495 kDa HA were used to determine HA molecular weight. **b**. Distribution of HA abundance (mean +/− SEM) by molecular weight size, determined by Select-HA: PBS (*n* = 3), LPS 1 dpi (*n* = 4), LPS 2 dpi (*n* = 3). **c**. Levels of HA ranging from 1000 to 2500 kDa (HMW) or 250–500 kDa levels were determined by integrating the area of HA abundance over the specified molecular weight ranges for each individual mice. Each data point represents an individual mouse lung. *n* = 3–4 mice per group. Data analyzed with ANOVA and Tukey’s multiple comparisons, *P < 0.05, **P < 0.01. HMW, high molecular weight; SEM, standard error of the mean; AU, arbitrary unit

Since HC-modified HA was present during early inflammation, but absent at later time points (i.e. day 4), we sought to determine whether there was breakdown of high molecular weight (HMW) HA. Accordingly, the abundance of HA fragments of various molecular weights in lung tissue were measured in LPS- or PBS exposed mice. When compared to control conditions, shortly following LPS injury (days 1 and 2), the abundance of HMW HA fragments (1000–2500 kDa) decreased, while the abundance of medium molecular weight HA fragments (250–500 kDa) increased (Fig. 3a-c). Of note, the increased appearance of medium molecular weight HA fragments in whole lungs following LPS exposure was similar in *TSG-6*-KO mice compared to littermate heterozygous control mice (Additional file 5: Figure A), suggesting that these fragments are generated independently of HC-modified HA. In parallel with these changes in whole lungs, we measured the total HA content in bronchoalveolar lavage, using ELISA (which detects HA fragments of all sizes with a minimum limit of detection between 6 KDa and 15 kDa [37]). These data showed increased HA levels at day 1 following LPS which returned towards baseline at days 4 and 6 (Additional file 5: Figure B). Similar to findings in whole lung, there was no significant difference between *TSG-6*-KO and control littermates in HA levels in the bronchoalveolar lavage (Additional file 5: Figure B).

**Fig. 4:**
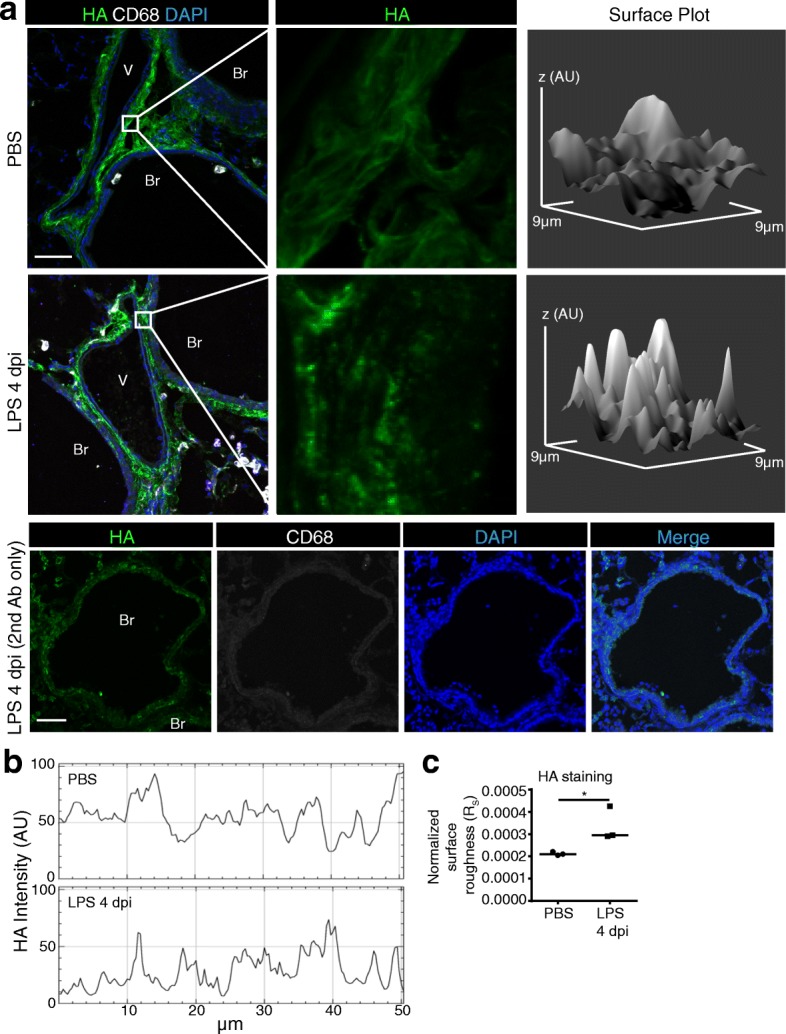
Roughness of peribronchial HA following LPS. **a**. Identification of HA staining in peri-broncho-vascular interstitial areas bordered by blood vessels (V) and bronchi (Br) in paraformaldehyde fixed, frozen sections of lungs instilled with LPS (4 dpi) or control (PBS) and immunostained with HA-binding protein (green), antibody against CD68 (macrophage marker, white), and the nuclear stain DAPI (blue). A negative control for staining, using secondary antibody only is shown in the bottom row. Surface plots of the maximum intensity of staining (Z-projection) spanning 9.4 × 9.4 μm areas were generated, shown to the right, measured in arbitrary units (AU); Scale bar 50 μm. **b**. Representative line profile of HA staining in the max intensity Z-projection (50 × 50 μm). **c**. The normalized surface roughness of HA staining intensity was determined for both PBS and LPS (4 dpi) treated lungs by dividing the surface area of the intensity plots by the average staining intensity (*n* = 3 mice per group); ANOVA with Tukey’s multiple comparisons; *P < 0.05)

To further quantify and localize HA remodeling, we adapted a previously described method [38], to detect HA immunofluorescence on paraformaldehyde-fixed frozen lung sections assessed with confocal microscopy. Compared to the lamellar pattern of HA staining seen in the peri-broncho-vascular regions of PBS-exposed control mice, mouse lungs exposed to LPS exhibited a more granular and rough HA staining (Fig. 4a, Additional file 6). To quantify this change in the pattern of HA staining, which is thought to represent HA remodeling driven by HA fragmentation and synthesis, we constructed intensity surface plots of HA staining and calculated their surface roughness (Fig. 4b-c). HA staining from LPS-exposed lung sections had significantly greater surface roughness than control sections (Fig. 4c).

**Fig. 5:**
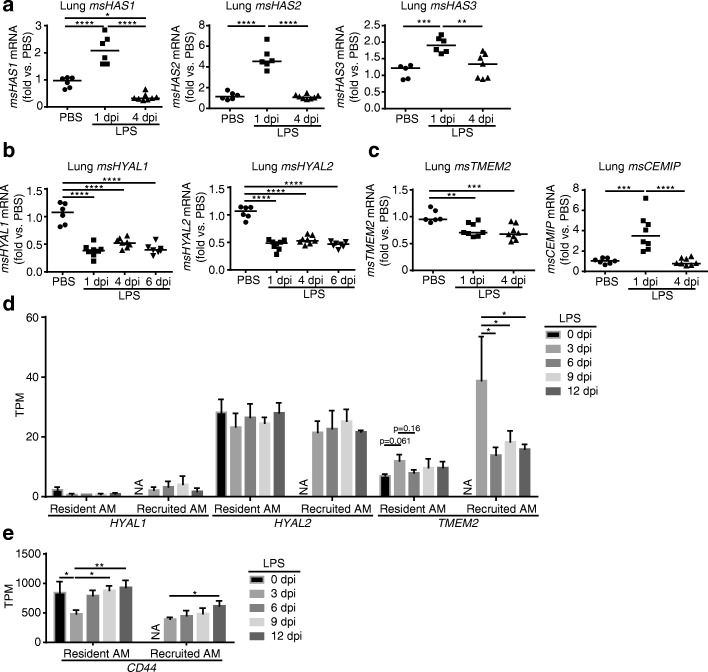
Effect of LPS on genes implicated in HA synthesis and breakdown in whole lung and alveolar macrophage. **a-c**. Expression of HA synthases (*HAS1–3,* **a**), hyaluronidases (*HYAL1–2,* **b**), transmembrane protein 2 (*TMEM2*) and cell migration-inducing and HA-binding *CEMIP* (**c**) was assessed by qPCR in lungs of mice instilled intratracheally with vehicle (PBS) or LPS for the indicated duration. Each data point represents an individual mouse. **d-e**. Expression of genes implicated in HA breakdown *HYAL1*, *HYAL2*, *TMEM2*, and CD44 in resident and recruited mouse alveolar macrophages isolated from bronchoalveolar lavage of mice treated with LPS (20 μg intratracheal; 0, 3, 6, 9, and 12 dpi). Expression level was assessed by RNA-seq and shown as transcripts per million (TPM). Mean (*n* = 3 independently pooled samples per time point, 4–7 mice for each pool) and SD plotted. NA, not applicable; SD, standard deviation. Data analyzed with ANOVA and Tukey’s multiple comparisons*;* *P < 0.05, **P < 0.01, ***P < 0.001, ****P < .0001

These results suggested HC-modification-independent HA fragmentation during ALI. Since HA synthases and hyaluronidases are major determinants of HA turnover, we measured their transcript abundance in lungs exposed to LPS. All three HA synthases (*HAS1–3)* were upregulated in mouse lungs at 1 day following LPS exposure, returning to baseline by day 4 (Fig. 5a). In contrast, the expression levels of key somatic tissue hyaluronidases (*HYAL1* and *HYAL2*) were persistently downregulated in murine lungs following LPS injury (for up to 6 days after instillation) (Fig. 5b). Of the two proteins recently implicated in HA breakdown that exhibit RNA expression in the lung [39–41], *TMEM2* (transmembrane protein 2) expression was also persistently downregulated; however, *KIAA1199/CEMIP* (cell migration inducing and hyaluronan binding protein) [40, 41] was significantly increased at 1 day following LPS exposure, returning to baseline by day 4 (Fig. 5c).

Since alveolar macrophages can bind and degrade HA [42], we additionally analyzed hyaluronidase expression in alveolar macrophage isolated from bronchoalveolar lavage using the RNA-seq database described above (Fig. 5d). In contrast to the whole lung, we noted a stable expression of *msHYAL1–2* and early induction of *msTMEM2* that was highest at day 3 in both resident and recruited alveolar macrophages. Compared to *msHYAL2* and *msTMEM2*, *msHYAL1* was expressed minimally and *msCEMIP* was not detected (one transcript per million threshold cut-off). Since HYAL2’s ability to degrade HA depends on CD44 binding of HA at the cell surface [43, 44], we assessed CD44 expression and noted that it was highly expressed and increased during resolution reaching peak expression on day 12 in both recruited and resident macrophages (Fig. 5e).

### Function of HC-HA formation during ALI

**Fig. 6:**
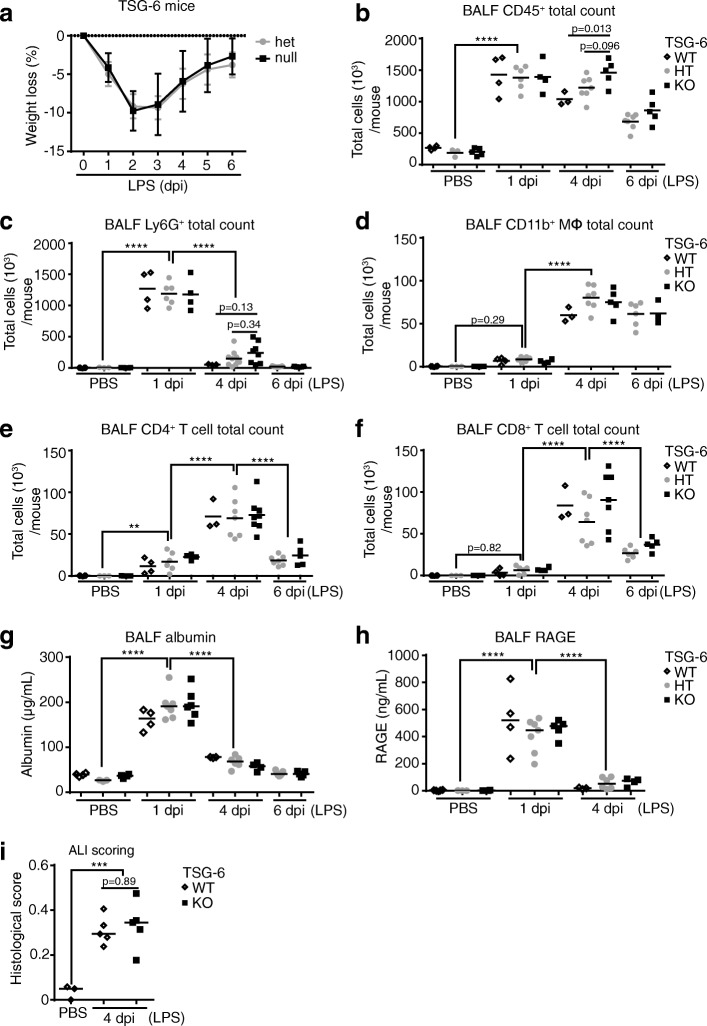
Effect of TSG-6 deficiency on severity and resolution of LPS induced ALI. **a**. Daily weight loss over six days following LPS instillation shown as % change from baseline (mean +/− SD). **b-f**. Total bronchoalveolar lavage counts of CD45^+^ leukocytes, Ly6G^+^ neutrophils, CD11b^+^ macrophages, CD4^+^ T-cells, and CD8^+^ T-Cells determined by flow cytometry following PBS or LPS instillation for the indicated time. **g-h**. Levels of albumin and RAGE in bronchoalveolar lavage were determined by ELISA for mice treated with PBS or LPS. **i**. Lung injury scores were calculated from hematoxylin and eosin stained lung sections to assess the rate of ALI resolution in *TSG-6* KO and WT mice treated with LPS. *n* = 3–7 per group. ANOVA with Tukey’s multiple comparisons. **P < 0.01, ****P < .0001. SD, standard deviation

HC-modified HA is critical for neutrophil sequestration in liver sinusoids during sepsis induced by systemic LPS exposures [6, 7]. We investigated the role of HC-HA formation on outcomes of lung injury induced by direct lung instillation of LPS in TSG-6 sufficient and TSG-6 deficient mice. *TSG-6*-KO mice exhibited similar body weight loss and recovery following lung instillation of LPS (Fig. 6a). To assess the impact of TSG-6 on lung inflammation, we measured leukocyte abundance in the airway and airspaces using flow cytometry on cells harvested by bronchoalveolar lavage. (Additional file 7). In response to lung instillation of LPS, *TSG-6*-KO mice had similar levels of total CD45^+^ leukocytes, neutrophils, CD11b^+^ macrophages, CD4^+^ T cells, and CD8^+^ T cells in the bronchoalveolar lavage as their littermate controls at almost all time points studied (Fig. 6b-f). Compared to wild type or heterozygous mice, CD45^+^ leukocytes were significantly or tended to be higher in *TSG-6*-KO mice only at day 4 following LPS. A similar trend was observed for neutrophils. To gauge the role of endogenous TSG-6 on the severity of lung injury in this model, we measured albumin and RAGE in the bronchoalveolar lavage as markers of endothelial and epithelial barrier integrity, respectively (Fig. 6g-h). Both markers were significantly elevated at day 1 following LPS injury, followed by return to baseline, to similar extent and kinetics in *TSG-6*-KO and littermate control mice. To confirm these findings, lungs at day 4 following LPS were formalin-fixed, paraffin-embedded, hematoxylin and eosin stained, and scored for lung injury severity (Fig. 6i, Additional file 8) using current guidelines [20]. Lung injury scores were not significantly different between *TSG-6*-KO and -WT mice.

**Fig. 7:**
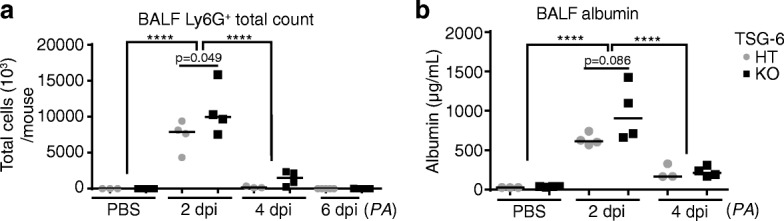
Effect of TSG-6 deficiency on severity and resolution of *PA* induced ALI. **a**. Total bronchoalveolar lavage counts of Ly6G^+^ neutrophils were determined by flow cytometry following PBS or LPS instillation at the indicated times. **b**. Level of albumin in bronchoalveolar lavage was determined by ELISA for mice treated with PBS or LPS. *n* = 3–4 per group. ANOVA with Tukey’s multiple comparisons. ****P < .0001

To extend our findings to gram negative bacterial infection, total neutrophil counts and albumin in bronchoalveolar lavage were similarly assessed following *PA* (Fig.7a-b). Compared to heterozygous littermates, lavage neutrophils and albumin were also significantly or tended to be higher in *TSG-6*-KO mice. Together with the data obtained using LPS, these results suggest a modest role for HC-HA formation in the onset, severity, or resolution of lung inflammation and injury during respiratory infection-induced ALI.

## Discussion

This study indicates that lung infections induce rapid covalent modification of HA, which was dependent on the evolutionarily conserved ability of TSG-6 to form HC-modified HA. In this context, alveolar macrophages may be an important source of secreted TSG-6 in the lung. Concomitant with HC-HA formation, we noted robust HA fragmentation which was independent of TSG-6. Both HC-modified HA and airway HA levels subsided with the resolution of lung injury. However, the TSG-6-dependent formation of HC-HA in this model did not have a major impact on the extent of lung inflammation and injury in this model.

Unlike other glycosaminoglycans (e.g. heparan sulfate, dermatan sulfate, chondroitin sulfate, and keratan sulfate), HA’s dissacharide backbone exhibits the least diversity, because HA cannot be covalently modified by sulfation, deacetylation, epimerization, and membrane-bound core proteins. Instead, the evolutionarily conserved TSG-6 mediated modification of HA’s N-acetylglucosamine with serum IαI’s HC is the only known HA covalent modification [5, 45]. Preclinical LPS-induced endotoxic shock models of sepsis showed that HC-modified HA was required for neutrophil sequestration in the liver [6, 7] and was overall protective for animal survival [8, 9], indicating that endogenous TSG-6 secretion is important for the control of bacteria-induced inflammation and injury. *TSG-6*- and *IαI*-KO mice not only had worse endotoxic shock outcomes [8, 9], but they also had markedly increased lung neutrophil infiltration [8, 46], suggesting a protective role for TSG-6-mediated formation of HC-HA in the lung in controlling local neutrophil recruitment and accumulation, and/or a major reduction in the pool of recruitable neutrophils to other organs due to liver sequestration and removal of circulating neutrophils [10].

Our data indicate a modest impact of TSG-6 on neutrophil levels in the bronchoalveolar lavage following lung instillation of LPS, suggesting that the lung production of TSG-6 may not be a major determinant of acute inflammatory cell accumulation, at least in ALI induced by lung rather than systemic endotoxin exposure. It is possible that in this more localized injury model, the levels of systemically absorbed LPS were insufficient to cause major formation of HC-modified HA in the liver with subsequent entrapment of neutrophils. In addition, since the HA expression in liver sinusoidal vasculature is 500- and 600-fold higher than in the lung vasculature both at baseline and during endotoxemia [7], much higher levels of TSG-6 than those locally produced in ALI may be required to significantly modulate inflammation, including neutrophil trafficking across lung tissue barriers. This notion is further supported by the remarkable anti-inflammatory effects following treatment with exogenous recombinant TSG-6 in several conditions, including models of ALI [8, 47]. These protective effects of exogenous TSG-6 have been ascribed to its ability to bind to and inhibit neutrophilic chemokines [48–50], and may also be linked to an effect on bone marrow myeloid progenitor cell function [22] and stromal cell differentiation [51–53].

Our report is the first to use knockout mice to investigate the specific role of TSG-6 and HC-modified HA during bacterial lung infection. We found that there are similar trends toward greater neutrophilic inflammation during TSG-6 deficiency in both LPS and gram negative bacterial infection models of localized lung injury (Figs. 6a-b and 7). Since these trends were observed at time points of inflammation that directly followed (4 dpi, LPS) or coincided with (2 dpi, *PA*) peak HC-HA levels, our data suggest that TSG-6 formation of HC-modified HA has modest effect on the abundance of inflammatory cells in the lung during acute infections. Considering the magnitude of the differences in BAL neutrophils and albumin levels, our results suggest that TSG-6 has a mild, protective role during acute lung inflammation. Given the well-established role of neutrophils in antibacterial defense [54–56], future studies are needed to carefully dissect whether induction of TSG-6 and HC-modified HA in the lung has a protective or deleterious role in eliminating the gram negative bacterial infection. Additionally, the role of TSG-6 during gram negative bacterial sepsis has not been investigated and remains unclear, since the published reports on the role of TSG-6 and its covalent modification of HA during sepsis have been performed using systemic administration of LPS.

Our investigations expand on previous studies that implicated PBMCs as sources of TSG-6 in response to TNFα or LPS [35, 36], by showing that terminally differentiated hAM are more versatile producers of TSG-6 compared to bronchoepithelial cells or lung fibroblasts. Furthermore, our comparative studies using adipose stem/progenitor cell suggested that hAM may be quite potent secretors of TSG-6 during acute inflammation. Locally produced TSG-6 may be essential for HC-HA formation in various lung compartments during bacterial or other types of inflammation associated with high TNFα levels, a cytokine implicated in the pathogenesis of a variety of acute and chronic lung diseases in humans [57–59]. The local levels of TSG-6 produced in the lungs were not major determinants of acute lung inflammation and injury following LPS instillation. The only significant impact of TSG-6 deficiency in our study was that of a persistent increase in total inflammatory cell counts in bronchoalveolar lavage, with a trend of affecting particularly neutrophils during the resolution of inflammation. The functional significance of this effect remains to be determined in chronic or irreversible models of lung injury. Given the lack of differences in acute lung injury indices in *TSG-6*-KO mice, we did not explore the role of TSG-6 on monocyte and macrophage function. This area has received recent attention in endotoxic shock models of systemic sepsis, where TSG-6 has been implicated in lung macrophage polarization [8, 60], attributed to its modulatory effects on HA interactions with its receptor CD44 on monocytes [61–63], or to a marked inflammatory milieu in *TSG-6*-KO mice that could also impact macrophage functionality and programming [8].

To our knowledge, this report is the first to characterize the kinetics of HC-HA formation and HA fragmentation and remodeling during LPS and *PA*-induced reversible lung injury in mice. We noted HC-HA covalent interactions followed by rapid clearance of HC-modified HA during lung inflammation, suggesting a high HA turnover during ALI, which resolves within 4 days after LPS intratracheal instillation. Ability to form HC-HA paralleled the increase in lung levels of *TSG-*6 and the availability of serum IαI (the source for HC) in the lung interstitium, as measured by correlations with markers of endothelial permeability (Additional file 9). The fact that the highest HC-HA levels coincide with peak alveolar permeability (BAL albumin and RAGE levels, 1 dpi) supports the hypothesis that vascular leak of serum-derived HC substrate into tissue is a critical step in the formation of HC-HA in lung tissue, as we have described before [14]. Since TSG-6 was not required for the control of lung injury and barrier function, it is possible HC-HA formation is not required for control of lung injury, but necessary for other processes that were not investigated, such as airway epithelial cell survival and homeostasis [64, 65].

Whereas HC-modified HA was not critical for the outcomes of ALI measured, future investigations will have to establish the functional role of HA fragmentation and remodeling in lung injury and repair. Unlike the accumulation and persistence of HC-modified HA observed in histopathological lesions of various chronic lung diseases, HC-modified HA in our models of ALI did not accumulate and was accompanied by markedly increased fragmentation of high molecular weight HA and/or de novo production of medium molecular weight HA products. Our study design could not differentiate between these two processes, nor did it carefully characterize the production of small molecular weight HA. The latter, however, were included in the total HA levels we measured in the bronchoalveolar lavage. These results suggest a key role of hyaluronidases in clearing HA to ensure resolution of acute lung inflammation, since hyaluronidase deficiency is associated with failure to clear HA and development of lung fibrosis [66]. However, the transcription of both *HYAL1* and *HYAL2,* which encode the principal hyaluronidases in human and mice [67] were decreased in ALI lungs. Although the actual hyaluronidase activity may diverge from the abundance of its mRNA [68], our data indicate that reactive oxygen species or other hyaluronidases may cause the marked HA fragmentation noted during ALI. We focused on two proteins that impact HA turnover whose genes are abundantly expressed in the lung: TMEM2 and KIAA1199/CEMIP [39–41]. Of these, the expression of *CEMIP*, a HA-binding protein that promotes HA degradation via clathrin-mediated endocytosis [39] in the whole lung paralleled the kinetics of HA fragmentation in ALI. In turn, in alveolar macrophages, it was the expression of CD44 that increased during the resolution phase of ALI, which may indicate a role in the proper clearance of HA fragments. Future studies should investigate the relative contribution of enzymatic vs. non-enzymatic (i.e. reactive oxygen species) regulation of HA turnover in ALI.

## Conclusions

Our study indicates that both HC-HA formation and HA degradation are rapidly and transiently induced in models of gram-negative bacterial respiratory infections that cause resolving ALI. The rapid HA turnover was associated with increased TSG-6 production, with induction of HA synthases expression, and with increased HA degradation-promoting *CEMIP* expression. While alveolar macrophages are likely sources of TSG-6 secretion following lung endotoxin exposure, the endogenous TSG-6-dependent HC-HA formation had a modest effect on reducing neutrophilic inflammatory cell abundance in the bronchoalveolar lavage during the resolving phases of ALI. TSG-6 is dispensable for the inflammatory response to transient ALI induced by lung infection, but its major role in forming HC-modified HA suggests that it may play a role in non-resolving inflammatory lung conditions associated with abnormal HA turnover.

## Additional files

Additional file 1: TSG-6 is conserved for catalyzing HC-modification of HA. **A**. Modifying HA with heavy chains (HC) of the serum protein inter alpha inhibitor (IaI), also known as serum-derived HA-associated protein (SHAP), is the only covalent modification HA can undergo. **B**. TNFα-stimulated gene-6 (TSG-6) is an inflammation-induced secreted protein that exclusively mediates formation of HC-modified HA. Through two transesterification reactions, TSG-6 transfers HC from IαI onto itself and then onto HA. To form the TSG-6-HC intermediate, HC is covalently linked to a conserved serine residue adjacent to the TSG-6 Link domain that binds HA and facilitates HC transfer [1–5]. The serum protein IaI consists of a chondroitin sulfate that is covalently linked to the light chain bikunin and two heavy chains (HC1 and HC2) that can be removed by TSG-6. **C**. Alignment of TSG-6 pro-protein (residues 1–47 depicted, hTSG-6 numbering) containing the signal peptide (highlighted in gray) and start of the HA-binding Link domain (highlighted in green). The serine residue (highlighted in yellow) responsible for removing HC from serum IaI and transferring HC onto HA is evolutionarily conserved across all vertebrates including fish, reptile, and bird: *Homo sapiens* (human), *Mus musculus* (mouse), *Equus caballus* (horse), *Bos taurus* (cattle), *Pelodiscus sinensis* (chinese softshell turtle), and *Danio rerio* (zebrafish). CLUSTAL multiple sequence alignment by MUSCLE 3.8 (MUltiple Sequence Comparison by Log-Expectation); “*” (asterisk) indicates fully conserved residue. “:” (colon) indicates residues with strongly similar properties (>0.5 in Gonnet PAM 250); “.” (period) indicates residues with weakly similar properties (≤0.5 in Gonnet PAM 250). (DOCX 95 kb)

Additional file 2: Human TSG-6 (hTSG-6) ELISA standard curves. hTSG-6 standard curves were obtained using recombinant hTSG-6 (R&D) in the absence and presence of FBS. FBS reduced the magnitude of HRP substrate color change. Of note, TSG-6 readily forms covalent complex with HC (TSG-6-HC intermediate, Fig. 2a-b) at a conserved serine residue (Additional file 1) in the presence of serum IαI source (e.g. FBS). Formation of the TSG-6-HC intermediate may sterically hinder the binding of TSG-6 by the capture and detection antibodies of TSG-6 sandwich ELISA. OD, optical density. (DOCX 43 kb)

Additional file 3: HC-modification of HA after LPS and *PA* injury. **A**. HC-modified HA following LPS injury at 1 and 4 dpi measured as in Fig. 1a. **B**. HC-modified HA following *PA* injury (2*10^6^ CFU) at 2 and 4 dpi measured as in Fig. 1b**.** HT denotes heterozygous *TSG-6* control littermate. (DOCX 110 kb)

Additional file 4: *TSG-6* expression in resident and recruited mouse alveolar macrophages. Resident and recruited mouse alveolar macrophages (msAM) were isolated from bronchoalveolar lavage of LPS treated mice (20 μg intratracheal; 0, 3, 6, 9, and 12 dpi). Expression level of *msTSG-6* was assessed by RNA-seq and shown as transcripts per million (TPM). Mean (*n* = 3 independently pooled samples per time point, 4–7 mice for each pool) and error bar (SD) plotted. ND, not detected; NA, not applicable; SD, standard deviation. (DOCX 42 kb)

Additional file 5: Effect of TSG-6 deficiency on lung HA molecular weight distribution and lavage HA levels following LPS injury. **A.** HA was extracted from lung tissue (LPS 1 dpi), separated by agarose gel, and visualized as described in Fig. 3. HT and KO denote *TSG-6* heterozygous and knockout mice. Select-HA consisting of 2500, 1000, 500, and 250 kDa HA was used to determine the molecular weight. **B.** HA levels in bronchoalveolar lavage were measured by ELISA. *n* = 5–10 mice per group. (DOCX 87 kb)

Additional file 6: Effect of LPS on HA staining. **A**. Representative images of paraformaldehyde-fixed, frozen lung sections immunostained as described for Fig. 4 shown here in detail. Changes in HA staining can be seen in the peri-broncho-vascular interstitium (white arrow). **B**. Representative sections from independent animals (*n* = 3 mice per group). Br depicts a bronchial airway and V pulmonary vessels. Scale bar 50 μm. (DOCX 821 kb)

Additional file 7: Schematic of flow strategy applied to lavaged cells from PBS and LPS treated mice. Bronchoalveolar lavage from mice at 4 day post LPS instillation is depicted to illustrate all the cell populations assessed. **A**. Total leukocytes were identified by excluding debris and doublets and using CD45^+^ staining. T cells were identified by CD3^+^ staining and differentiated by CD4^+^ and CD8^+^ staining. Neutrophils were identified by Ly6G^+^CD64^−^ staining. Macrophages were identified by CD64^+^F4/80^+^ and classified as recruited (CD11b^+^CD11c^low^) or resident (CD11b^low^CD11c^+^SiglecF^+^). **B**. Counting beads were identified by high SSC and low FSC and high fluorescence in FITC and PE. (DOCX 154 kb)

Additional file 8: Histologic scoring of ALI lungs**. A.** Formalin-fixed, paraffin-embedded mice lungs (4 day post LPS instillation) were stained with hematoxylin and eosin and scored at high power fields (400X magnification). Neutrophils marked with blue arrowhead. **B**. Representative images of lung sections from LPS injured *TSG-6* KO and WT mice compared to PBS control taken at lower power fields (100X and 200X). Scale bars: 25 μm (400X), 50 μm (200X), and 100 μm (100X). (DOCX 984 kb)

Additional file 9: Correlation between HC-HA and markers of alveolar permeability**.** Correlation plot of HC-HA abundance, measured in arbitrary units (AU) versus BAL fluid albumin (**A**) or RAGE (**B**) levels in individual mice exposed to LPS for 1 or 4 days compared to PBS control with simple linear regression line and coefficient of determination (R-squared). (DOCX 36 kb)

## Abbreviations

ALI: Acute lung injury
AM: Alveolar macrophage
ASC: Adipose stromal/progenitor cell
AU: Arbitrary unit
BALF: Bronchoalveolar lavage fluid
BSA: Bovine serum albumin
C: Celsius
cDNA: Complementary DNA
CFU: Colony forming unit
DAPI: 4′,6-diamidino-2-phenylindole
DMEM: Dulbecco’s Modified Eagle Medium
DNAse: Deoxyribonuclease
dpi: Day(s) post-instillation
*E. coli*: *Escherichia coli*
EBM2: Endothelial growth basal medium
ECM: Extracellular matrix
EDTA: Ethylenediaminetetraacetic acid
EGM2-MV: Endothelial cell growth medium
ELISA: Enzyme-linked immunosorbent assay
FBS: Fetal bovine serum
g: Gram
h: Hour
HA: Hyaluronic acid (hyaluronan)
hAM: Human alveolar macrophages
HAS: HA synthase
HC: Heavy chain (IαI)
HMW: High molecular weight
hPBDM: Human PBMC derived macrophage
HSPC: Hematopoietic stem and progenitor cell
HT: Heterozygous
hTSG-6: Human TSG-6
HYAL: Hyaluronidase
IACUC: Institutional Animal Care and Use Committee
IαI: Inter-alpha-inhibitor
kDa: kiloDalton
KO: Knockout
LB: Lysogeny broth
LPS: Lipopolysaccharide
M: Molar concentration
mAM: mouse alveolar macrophage
MEM: Minimum essential media
mg: milligram
mL: milliliter
mM: millimolar
mRNA: messenger RNA
MSC: Mesenchymal stem cell
msCEMIP: Mouse cell migration inducing hyaluronan binding protein
msTSG-6: Mouse TSG-6
NCBI: National Center for Biotechnology Information
ng: nanogram
OCT: optimal cutting temperature
*PA*: *Pseudomonas aeruginosa*
PBDM: PBMC derived macrophage
PBMC: Pperipheral blood mononuclear cell
PBS: Phosphate buffered saline
PFA: Paraformaldehyde
pH: Potential of hydrogen
qPCR: Quantitative polymerase chain reaction
qPCR: Quantitative real-time polymerase chain reaction
RAGE: Receptor for advanced glycation end products
RNA: Ribonucleic acid
RNA-Seq: RNA-sequencing
RPMI: Roswell Park Memorial Institute
SD: Standard deviation
SDS-PAGE: Sodium dodecyl sulfate polyacrylamide gel electrophoresis
siRNA: Small interfering RNA
TMEM2: Transmembrane protein 2
TNFAIP6: Tumor necrosis factor-inducible gene 6 protein
TNFα: Tumor necrosis factor α
TRU: Turbidity reducing units
TSG-6: TNFα-stimulated gene-6
U: Unified atomic mass *unit*
WT: Wild type
ΔΔCt: Comparative CT
μg: Microgram
μL: Microliter
μm: Micrometer
