# Supplementary material for: Rapid clearance of heavy chain-modified hyaluronan during resolving acute lung injury
Source: Respir Res. 2018 May 31;19:107. doi: 10.1186/s12931-018-0812-1 (PMC5984366; doi:10.1186/s12931-018-0812-1)

Additional File 1

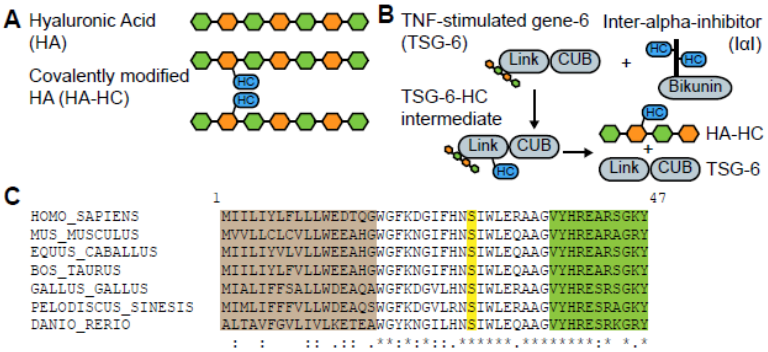

Supplement: Supplementary file 1 [file 12931_2018_812_MOESM1_ESM.docx]
